# Gut microbiota and allogeneic transplantation

**DOI:** 10.1186/s12967-015-0640-8

**Published:** 2015-08-23

**Authors:** Weilin Wang, Shaoyan Xu, Zhigang Ren, Jianwen Jiang, Shusen Zheng

**Affiliations:** Department of Hepatobiliary and Pancreatic Surgery, First Affiliated Hospital, Zhejiang University School of Medicine, 79 Qingchun Road, Hangzhou, 310003 Zhejiang China; Key Laboratory of Combined Multi-organ Transplantation, Ministry of Public Health, First Affiliated Hospital, Zhejiang University School of Medicine, 79 Qingchun Road, Hangzhou, 310003 Zhejiang China; Collaborative Innovation Center for Diagnosis and Treatment of Infectious Diseases, First Affiliated Hospital, Zhejiang University School of Medicine, 79 Qingchun Road, Hangzhou, 310003 Zhejiang China

**Keywords:** Transplantation, Gut microbiota, Immune system, Complication, Antibiotic, Probiotics, Prebiotics

## Abstract

The latest high-throughput sequencing technologies show that there are more than 1000 types of microbiota in the human gut. These microbes are not only important to maintain human health, but also closely related to the occurrence and development of various diseases. With the development of transplantation technologies, allogeneic transplantation has become an effective therapy for a variety of end-stage diseases. However, complications after transplantation still restrict its further development. Post-transplantation complications are closely associated with a host’s immune system. There is also an interaction between a person’s gut microbiota and immune system. Recently, animal and human studies have shown that gut microbial populations and diversity are altered after allogeneic transplantations, such as liver transplantation (LT), small bowel transplantation (SBT), kidney transplantation (KT) and hematopoietic stem cell transplantation (HTCT). Moreover, when complications, such as infection, rejection and graft versus host disease (GVHD) occur, gut microbial populations and diversity present a significant dysbiosis. Several animal and clinical studies have demonstrated that taking probiotics and prebiotics can effectively regulate gut microbiota and reduce the incidence of complications after transplantation. However, the role of intestinal decontamination in allogeneic transplantation is controversial. This paper reviews gut microbial status after transplantation and its relationship with complications. The role of intervention methods, including antibiotics, probiotics and prebiotics, in complications after transplantation are also discussed. Further research in this new field needs to determine the definite relationship between gut microbial dysbiosis and complications after transplantation. Additionally, further research examining gut microbial intervention methods to ameliorate complications after transplantation is warranted. A better understanding of the relationship between gut microbiota and complications after allogeneic transplantation may make gut microbiota as a therapeutic target in the future.

## Background

There are more than one thousand microbial species living in the complex human gut ecosystem and most of these species are bacteria [1]. The microbial density in fecal matter is approximately 10^13^ to 10^14^ cells/g with 70 % of the total microbes in the colon [2]. Microbial communities in the gut are important in protecting the host against pathogenic microbes [3–5] as well as regulating metabolic processes [6, 7], and have been regarded as peacekeepers [8] as well as a neglected endocrine organ [9]. Notably, gut microbiota can drive the maturation of host immune system [10]. It plays important roles in the normal architecture of secondary lymphoid organs, differentiation of induced regulatory T cells (iTregs) and generation of immunoglobulin A (IgA)-secreting B cells. However, gut microbial dysbiosis is associated with the development of inflammatory bowel disease [11, 12], obesity [2], diabetes [13, 14], colorectal cancer [15, 16], liver diseases [17], cardiovascular disease [18], nervous system diseases [19], etc.

Classical studies of gut microbiota are largely dependent on culturing techniques, which can only culture 10–30 % of gut microbiota [20–22]. In recent years, the rapid development of advanced molecular technologies, such as polymerase chain reaction-denaturing gradient gel electrophoresis (PCR-DGGE), and next-generation sequencing (NGS) technologies, including 16S rRNA sequencing [23, 24] and metagenomic sequencing [25], has facilitated the analysis of a large number of microorganisms in the gut.

Allogeneic transplantation is a potentially curative therapy for a large number of end-stage diseases. However, complications after transplantation, such as infections, rejection, graft-versus-host disease (GVHD) and relapse, remain challenges of its widespread use [26–30]. Moreover, infections have also been associated with episodes of acute and chronic rejection [31]. It is usually thought that tissue microbiota has a major influence on local immunity. However, gut microbiota is also thought to impact distal immune responses and modulate diseases in distant tissues in conditions, such as liver diseases [17], cardiovascular disease [18], rheumatoid arthritis and obesity. Thus, alloimmune responses to transplanted organs may also be influenced by gut microbiota. In recent years, many animal and human studies have indicated that gut microbial dysbiosis is closely linked with allogeneic transplantation, such as liver transplantation, small bowel transplantation, kidney transplantation and hematopoietic stem cell transplantation, and especially with post-transplantation complications.

## Gut microbiota and the immune system

**Fig. 1:**
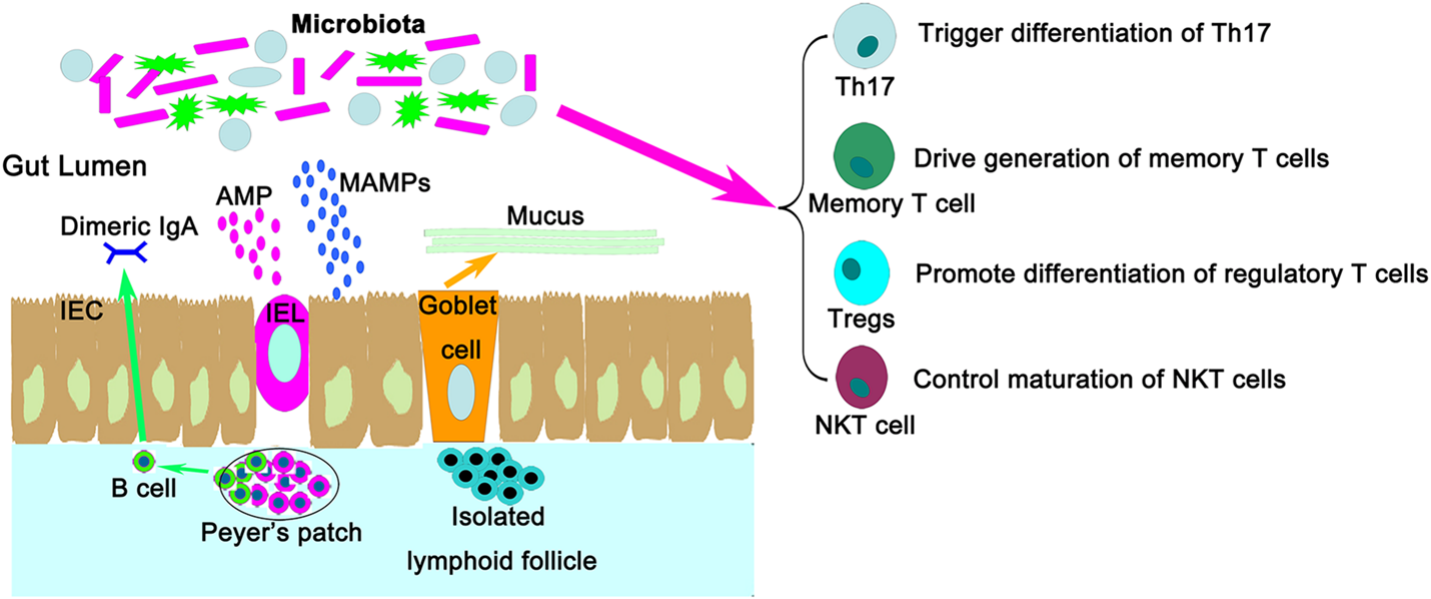
The interplay between gut microbiota and host’s immune system. Host’s immune system keeps gut microbiota stable and prevent outgrowth of pathogenic species by production of antimicrobial peptides (AMP) creating a sterility gradient, mucus separating the microbiota from the host, and secretory IgA neutralizing biologically active antigens. Gut microbiota are also important to the generation of optimal immune responses, including triggering differentiation of Th17 and regulatory T cells, driving generation of memory T cells and controlling maturation of NKT cells. *MAMPs* microbial-associated molecular patterns, *IEL* intraepithelial lymphocyte, *IEC* intestinal epithelial cell and *Treg* T regulatory cell

A large number of studies have shown that post-transplantation complications are closely related with the immune system [32–34]. To clarify the relationship between gut microbiota and allogeneic transplantation, it is very important to discuss the interplay between gut microbiota and the host’s immune system [35, 36] (Fig. 1).

It has been proven that the intestinal immune system can maintain gut bacteria homeostasis and prevent dysbiosis (Fig. 1). Epithelial, mucosal and immune cells at barrier surfaces of the intestinal tract all are important in maintaining gut microbial homeostasis and modulating microbes by producing mucus, antimicrobial peptides or luminal immunoglobulins. Some immune cells are intercalated between intestinal epithelial cells (IECs), such as intraepithelial lymphocytes (IELs), or underneath the epithelium, such as lamina propria immune cells. Others are organized into intestinal lymphoid structures, including isolated lymphoid follicles (ILFs), Peyer’s patches (PPs) and mesenteric lymph nodes (MLNs). Impairment or lack of these immune structures may lead to gut microbial dysbiosis. For example, Gram negative bacteria were over-represented in mice lacking ILFs [37].

Gut microbiota is also important to a host’s immune system. In transplantation, T cells are important in transplant rejection. Interestingly, several studies found that specific gut bacteria species can promote T cell differentiation. In rats, Th17 cell differentiation can be stimulated by Segmented filamentous bacteria (SFB) [38] and *Lactobacillus johnsonii* [39]. Gut microbiota may also contribute to the generation of memory alloreactive T cells. Hand et al. [40] found that, during a gastrointestinal infection, both the pathogen and intestinal commensal bacteria could cause immune responses and lead to commensal-reactive T-cell memory. Anticommensal T-cell memory may result in a pool of memory cells with cross-reactive T-cell receptors (TCRs). In addition, several gut microbe species have been shown to promote expansion or differentiation of forkhead box protein 3 (Foxp3)-expressing regulatory T cells (Tregs). Some of these colonic Tregs recognize microbial antigens [41, 42]. Additionally, colonic Tregs are increased in germfree mice with a set of defined benign commensals termed altered Schaedler flora [43]. Indigenous *Clostridium* species have the potential to promote colonic inducible Treg (iTreg) differentiation [44]. Moreover, commensal gut microbiota can also control the development and maturation of mucosal and systemic natural killer T cells (NKTs) [45] and help the development and maturation of lymphoid structures [46].

Collectively, these data indicate that gut microbiota can interact with the immune system. Determining the relationship between gut microbiota and transplant complications, including infections, rejection, GVHD and relapse after transplantation, is urgent.

## Gut microbiota and allogeneic transplantation

In recent years, the progress of microbial detection technologies has facilitated studies evaluating the relationship between gut microbiota and allogeneic transplantation. Many animal experiments and human studies have shown that gut microbiota is altered after allogeneic transplantation. When postoperative complications occur, gut microbiota populations and diversity are in a more significant dysbiosis (Table 1).

**Table 1:** Changes of gut microbiota in complications after transplantation

	Complications	Changes in microbiota	Animal/human studies
LT	Acute rejection	*Bacteroides and Ruminococcus* ↑ [51]	Animal study
		Phylum Bacteroidetes ↑ phylum Firmicutes *↓* [52]	Animal study
	Infection	*Bifidobacterium dentium* ↑ [53]	Human study
	Chronic bile duct hyperplasia	*Enterococcus and Enterobacteria * ↑*Bifidobacterium and Lactobacillus *↓ [56]	Animal study
SBT	Acute rejection	Phylum Proteobacteria ↑ phylum Firmicutes ↓ [61]	Human study
	Chronic rejection	*Escherichia coli, Bacteroides* spp. and *Clostridium* spp. ↑ Lactobacillales ↓ [62]	Animal study
KT	Diarrhea	*Bacteroides, Ruminococcus* and *Coprococcus* ↓ [63]	Human study
	Urinary tract infection	*Enterococcus* ↑ [63]	Human study
	Acute rejection	*Bacteroidetes* ↓ [63]	Human study
HSCT	Graft-versus-host disease	*Enterococci* ↑ [66]	Human study
		Lactobacillales ↑ Clostridiales ↓ [69]	Animal study
		*Escherichia coli* ↑ [70]	Animal study
		*Enterococci and Bacteroides/Prevotella* spp. ↑ [78]	Animal study

*LT* liver transplantation, *SBT* small bowel transplantation, *KT* kidney transplantation and *HSCT* hematopoietic stem cell transplantation

### Liver transplantation

Thus far, the gut microbial status after LT has been mostly studied in animals. To investigate intestinal microbial levels and bacterial translocation (BT) following LT, Yu et al. [47] performed a study on male Brown-Norway (BN) rats. They found that the number of *Bifidobacterium* and *Lactobacillus* in the feces was markedly decreased in rats following a LT. However, *Enterobacteriaceae* and *Enterococcus* counts were significantly increased compared with rats without a LT. Moreover, the incidence of BT to the liver, spleen and mesenteric lymph nodes after the LT was increased. Recently, due to the development of technologies to detect gut microbiota, the characteristics of gut microbiota after LT are more accurate. Using PCR-DGGE, Xie et al. [48] found a similar result as Yu et al. [47] in Sprague–Dawley rats. Moreover, one month after orthotopic LT (OLT), the microbial alteration did not completely return to normal in cirrhotic rats. In studying fresh feces samples from participants in China, real-time quantitative PCR data of six interesting gut bacteria showed that *Eubacteria*, *Bifidobacterium* spp., *Faecalibacterium prausnitzii* and *Lactobacillus* spp. were significantly decreased following the LT and that *Enterobacteriaceae* and *Enterococcus* spp. were significantly increased [49]. Over time after LT, bacteria, except for *Enterococcus* spp., showed the potential to restore to normal. [49]. A later study [50] found that the fecal *Lactobacilli* population in patients with hepatitis B cirrhosis treated with LT was simpler than in healthy people.

Acute rejection (AR) and infection remain life-threatening complications after LT. In recent years, gut microbial features after LT with complications have been evaluated in several studies. A study that monitored gut microbial alteration in rats after an OLT using PCR-DGGE showed that the gut microbiota in rats with AR after an OLT was dominated by *Bacteroides* and *Ruminococcus* overgrowth. These changes were associated with elevated plasma endotoxin and a higher rate of BT [51]. By dynamically detailing the intestinal microbial characterization with PCR-DGGE, Ren et al. [52] analyzed gut microbiota of ileocecal contents in rats following an OLT. They found that microbial populations and diversity were decreased during AR with a decrease in phylum *Firmicutes* and increase in phylum Bacteroidetes. Lu et al. [53] prospectively analyzed the predominant intestinal microbiota of 12 patients before LT and at three weekly postoperative follow-up visits within the first month. Their DGGE profile results showed that patients with an infection had a substantial decrease of intestinal microbial diversity. Moreover, fecal DGGE profiles of two patients who had infections showed *Bifidobacterium dentium*, which is reported to mainly survive in the oral cavity [54] but is able to survive in an abnormal intestine [55]. Compared to a normal group, rats with chronic bile duct hyperplasia after allogeneic liver transplantation had remarkably reduced numbers of *Bifidobacterium* and *Lactobacillus*, whereas *Enterococcus and Enterobacteria* were significantly increased [56]. Intestinal bacteria also had a link with increased ischemia/reperfusion injury assessed by transaminase expression in a mouse model of OLT [57]. Flagellin, a TLR5 agonist, can be shed by gut bacteria and up-regulated intercellular adhesion molecule 1 on hepatic sinusoidal endothelium. As a result, liver-derived Kupffer cells are activated. Kupffer cell proliferation and MHC Class II expression are then enhanced. Phagocytic activity is suppressed and results in enhanced ischemia/reperfusion injury [57].

### Small bowel transplantation

Recently, studies were also performed to study the relationship between gut microbiota and small bowel transplant. Hartman et al. [58] used qPCR to assay the bacterial population in the small bowel lumen over time in 17 small bowel transplant patients. Surprisingly, the post-transplant microbial community was dominated by *Lactobacilli* and *Enterobacteria*, which are both typically facultative anaerobes. This is significantly different from normal colonization, which is dominated by the strict anaerobes *Bacteroides* and *Clostridia*. They also found *Lactobacilli* and *Enterobacteria* in patients with ileostomies who had not received a transplant. However, after surgical closure of the ileostomy, colonization reverted to the normal strict anaerobes. Thus, the authors suggested that an ileostomy itself might be a primary ecological determinant in shaping microbiota. Additionally, they indicated that there was robust small bowel ecological resilience after SBT. Fungi form a diverse microbial community in the human intestine. Little is known about the succession of species after SBT. Li et al. [59] initially reported temporal alterations in fungal communities in patients after an intestinal allograft. DGGE data showed that *Saccharomyces cerevisiae* and *Kluyveromyces waltii* dominated the fungal microbiota in patients with a SBT. Some species, including *Candida* spp., *Cryptococcus neoformans*, *Fusarium oxysporum*, *Aspergillus clavatus* and *Trichophyton verrucosum*, were present early after the SBT. These results may provide novel insight into the roles of the fungal microbiota in the pathophysiology of the transplanted intestine.

There is a close relationship between gut microbial dysbiosis and complications, such as GVHD and rejection, after SBT. In a heterotopic rat model following SBT, Price et al. [60] found that rejection and GVHD were associated with shifts in gut microflora toward potentially pathogenic organisms (*Staphylococcus epidermidis*) and bacterial translocation into recipient tissues posed a major threat for the development of sepsis. By pyrosequencing 16S ribosomal RNA gene tags, Oh et al. [61] indicated that, during episodes of rejection after SBT, the proportions of phylum Firmicutes and order Lactobacillales in ileal effluents were significantly decreased. However, those of phylum Proteobacteria, especially the family Enterobacteriaceae, were significantly increased. A receiver-operating characteristic analysis revealed that the presence of Firmicutes could be used to discriminate between non-rejection and active rejection. A DGGE analysis of the luminal and mucosal microbiota compositions in chronic rejection (CR) rats 190 days after SBT revealed that the gut microbiota in the CR rats had a decrease in the abundance of Lactobacillales bacteria, but an increase in *Escherichia coli*, *Bacteroides* spp. and *Clostridium* spp. [62].

### Kidney transplantation

Very recently, the gut microbial characteristics after kidney transplantation have been shown in two clinical studies. Lee et al. [63] prospectively enrolled 26 kidney transplant recipients and collected serial fecal specimens during the first 3 months after transplantation. Fecal microbial composition was identified using a PCR amplification of the 16S rRNA V4–V5 variable region and deep sequencing using the Illumina MiSeq platform. As a result, compared to pre-transplantation specimens, Proteobacteria was increased in specimens after kidney transplantation. In patients with post-transplant diarrhea, the diversity of fecal microbiota was lower than those without diarrhea. In addition, *Bacteroides*, *Ruminococcus* and *Coprococcus* were significantly lower in the patients with diarrhea. A principal coordinate analysis revealed significant differences in fecal microbial composition between the AR and non-AR groups. Urinary tract infections with *Enterococcus* and fecal abundance of *Enterococcus* were also noted. Another clinical study [64] also noted specific differences in pre-transplant microbiota of rectal samples during rejection events and infectious complications after transplantation. Rejection events were correlated with significant decreases in *Anaerotruncus, Coprobacillus, Coprococcus* and an unknown member of the Peptostreptococcaceae (all from phylum Firmicutes) in four patients compared to 14 patients without adverse events post-transplant. In the four patients with post-transplant infections, the genus *Anaerotruncus* (phylum: Firmicutes) was markedly decreased compared to 14 control samples. These findings suggest that specific microbiota features have the potential to be markers to predict patient history even before transplantation. In addition, in rectal swab samples, more significant microbial changes were observed between pre-transplant and 1-month post-transplant time points, than between 1-month and 6-month post-transplant time points. Moreover, Firmicutes accounted for the majority of bacterial genera, with significant changes between pre-transplant and 1-month post-transplantation time points.

### Hematopoietic stem cell transplantation

Autologous or allogeneic hematopoietic stem cell transplantation (HSCT) is a potentially curative treatment for various malignant and nonmalignant disorders. Bone marrow transplantation (BMT) is a type of HSCT. Graft-versus-host disease (GVHD), infections and relapse remain the major complications of HSCT and remain huge challenges for more widespread and effective use of this potent therapy.

Several studies revealed that HSCT was related to gut microbial alterations. In one study, intestinal microbiota of patients was characterized using 454 pyrosequencing of bacterial 16S ribosomal RNA genes. During allo-HSCT, the diversity and stability of the intestinal microbiota were disrupted and became dominated by bacteria associated with subsequent bacteremia [65]. Using next-generation sequencing technology, a relative shift toward *Enterococci* was observed in stool specimens after transplantation, which was more pronounced with antibiotic prophylaxis and treatments for neutropenic infections [66]. At the time of admission, patients showed a predominance of commensal bacteria. Using 454-pyrosequencing of 16S rRNA genes, Montassier et al. [67] first observed a significant reduction in alpha diversity and marked differences in the composition of intestinal microbiota in response to chemotherapy. Chemotherapy was associated with a drastic drop in *Faecalibacterium* and increase in *Escherichia*. Mortality outcomes seem to be related with gut microbiota because patients with significantly worse mortality outcomes had lower gut microbial diversity [68].

Several studies have shown that gut microbial dysbiosis may have a link with complications after HSCT, including GVHD. In mouse models and patients with GVHD after BMT, Jenq et al. [69] observed a loss of microbial diversity and Clostridiales and expansion of Lactobacillales in intestinal microbiota. Eliminating Lactobacillales from the gut flora in mice before BMT could cause GVHD. When reintroducing a predominant species of *Lactobacillus*, GVHD was alleviated. After HSCT, a relative shift toward *Enterococci* in intestinal microbial communities was also found. Specifically, the shift was prominent in patients who subsequently developed or suffered from active gastrointestinal GVHD [66]. In another study, mice with GVHD lost microbial diversity and overwhelmingly expanded otherwise rare bacteria *Escherichia coli*. There was a close correlation between alterations in the intestinal microbiota and GVHD severity [70].

## Gut microbiota, complications and the immune system

As discussed above, gut microbial dysbiosis and complications after transplantation coexist. However, the role of a host immune system in the correlation between gut microbial dysbiosis and complications is rarely studied. Similar to pathogens, gut microbes express microbial-associated molecular patterns (MAMPs) such as lipopolysaccharides (LPS) that can be sensed by specialized receptors on various cells, including immune and gut endothelial cells, to communicate with the immune system. There are a variety of pattern recognition receptors (PRRs). The most studied ones are Toll-like receptors (TLRs) and NOD-like receptors [71]. Intracellular adaptors are indispensable in transferring PRR signaling information and MyD88 is the most studied one, which is a molecule downstream of all TLRs except TLR3. After being exposed to MAMPs, PRR signaling in cells can promote the expression of major histocompatibility complex (MHC) and costimulatory molecules, particularly on antigen-presenting cells (APCs) and some endothelial cells [72]. As a result, cytokines such as tumor necrosis factor (TNF), type I interferons (IFNs), interleukin-1 (IL-1) and IL-6 are produced.

The liver can maintain tolerance against harmless antigens derived from commensal bacteria, even if commensal bacteria escape from the gut [73]. However, this surveillance could be temporarily perturbed after liver transplantation. After liver transplantation and during AR, loss of intestinal microvilli, tight junction damage, decrease in fecal secretory IgA and increases in blood bacteremia, endotoxin, and TNF-α were detected, along with dysbiosis of gut microbiota [52]. Furthermore, acute rejection of small intestine allografts was associated with increased TLR expression [74].

There is growing evidence that bacteria and innate PRRs are critically involved in the pathogenesis of acute GVHD after allogeneic stem cell transplantation. In experimental models, reduced GVHD severity, preserved graft-versus-leukemia effects and improved overall survival were found in allo-HSCT recipients that were treated with either anti-endotoxin neutralizing antibodies [75, 76] or an oral LPS inhibitor [77]. Alpha-defensins can selectively kill noncommensal microbes, but preserve commensal ones. However, Eriguchi et al. [70] discovered that Paneth cells were targeted by GVHD, which resulted in an obvious reduction in the expression of alpha-defensins. Moreover, Heimesaat et al. [78] analyzed both gut microbiota composition and impact of bacterial sensing via TLRs in intestinal GVHD (iGVHD). When iGVHD occurred after HSCT, gut microbiota shifted towards *Enterobacteria*, *Enterococci* and *Bacteroides*/*Prevotella* spp. An analysis of iGVHD in MyD88(−/−), TRIF(−/−), TLR2/4(−/−), and TLR9(−/−) recipient mice showed that bacterial sensing via TLRs was essential for iGVHD development. Increasing numbers of apoptotic cells, proliferating cells, T cells and neutrophils were found within the colons of mice with acute iGVHD. However, compared with wild-type controls, these responses were markedly reduced in MyD88 (−/−), TLR2/4(−/−), TRIF(−/−) and TLR9(−/−) mice. Meanwhile, TLR9(−/−) mice had increased survival rates, whereas TRIF(−/−) and TLR2/4(−/−) mice were not protected from mortality. These results not only emphasize the critical role of gut microbiota, innate immunity and TLR9 in iGVHD but also highlight an anti-TLR9 strategy as a potential novel therapy for iGVHD after HSCT. However, TLR-4 can be activated by MAMPs, especially LPS, and was found to be critical in inducing tissue protective factors and for protection against intestinal cell apoptosis during acute GVHD [79].

## Antibiotics, probiotics and prebiotics

To prevent or treat complications and ameliorate the imbalanced gut microbiota after allogeneic transplantation, gut microbial intervention methods are used. Antibiotics, probiotics and prebiotics are most often used. Promising and encouraging results have been obtained (Table 2). However, the role of intestinal decontamination in allogeneic transplantation is still controversial.

**Table 2:** The results of different gut microbial intervention methods

	Intervention methods	Results	Animal/human study
LT	SDD [81–85]	Reducing the high incidence of infection [81]	Human study
		Gram-positive microorganisms infection predominated over Gram-negative rods and anaerobes [82]	Human study
		No infection prevention [83–85]	Human study
	Antibiotics [57]	Partly ameliorating enhanced ischemia/reperfusion injury	Animal study
	LAB and fibers [87]	Reducing bacterial infection rates	Human study
	Only fibers [87]	Reducing incidence of severe infections	Human study
	Probiobics [88, 89]	Promoting partial restoration of intestinal microflora and improving intestinal barrier function [88]	Animal study
		Reducing the liver injury by acute rejection [89]	Animal study
SBT	Probiotics [90]	Ameliorating small bowel histological injuries and reducing BT	Animal study
HSCT	TGID [86]	Preventing acute GVHD	Human study
	Polymyxin B [70]	Ameliorating GVHD	Animal study
	Probiotics [91]	Reduceing acute GVHD and improving survival	Animal study

*LT* liver transplantation, *SBT* small bowel transplantation, *HSCT* hematopoietic stem cell transplantation, *KT* kidney transplantation, *LAB* actic acid bacteria, *SDD* selective digestive decontamination and *TGID* total gastro-intestinal decontamination

Colonization of the intestinal tract by various microbiota precedes infection in many cases, including LT. That lead to the evolution of selective digestive decontamination (SDD), which is initially described by Stoutenbeek et al. [80]. SDD aims to reduce the Gram negative and yeast flora in the gastrointestinal tract using antibiotics and antifungals to prevent infections. An early study [81] suggested that SDD could significantly reduce Gram negative aerobic bacteria and *Candida* colonization in the gut. It appeared to reduce the high incidence of infection related to these organisms in the early post-transplant period. However, in another study, after performing an SDD with norfloxacin, thirty-two patients had at least one episode of a major bacterial infection. Furthermore, the number of Gram positive microorganisms was greater than that of Gram negative rods and anaerobes [82]. Zwaveling et al. [83] found that SDD did not prevent infections in patients undergoing an elective LT. However, it did affect the type of infection. It demonstrated that Gram positive *cocci* infections replaced Gram negative *bacilli* and *Candida* species infections. Two recent studies showed that the use of SDD prophylaxis in LT patients affected the rate or distribution of infectious complications, duration of hospitalization, antibiotic use, or acquisition of resistant bacteria [84, 85]. However, enhanced ischemia/reperfusion injury assessed by transaminase expression in a mouse model of LT with down-regulated MAMP expression could be partly ameliorated using an antibiotic treatment [57]. Studies also found that GVHD after allogeneic BMT could be ameliorated by eliminating facultative and strict anaerobic microorganisms from the gastrointestinal tract with antimicrobial drugs in the period of time around the allogeneic BMT. Total gastrointestinal decontamination (TGID) was used by Vossen et al. [86] with high doses of non-absorbable antimicrobial drugs while the graft recipient was maintained in strict protective isolation. As a result, a successful TGID of the graft recipient prevented the development of acute GVHD after BMT. In another study, oral administration of polymyxin B could inhibit *Escherichia coli* outgrowth. Importantly, GVHD after HSCT was ameliorated [70].

Taking probiotics and prebiotics to regulate gut microbiota and reduce the incidence of complications after LT was also reported in several studies. Early enteric nutrition supplemented with a mixture of lactic acid bacteria and fiber reduced bacterial infection rates following LT. Treatment with only fiber led to a low incidence of severe infections [87]. In a BN rat study, Ren et al. [88] found that supplementation with probiotics, including *Bifidobacterium* and *Lactobacillus*, and long-term antibiotics promoted partial gut microbial restoration and improved intestinal barrier function in malnourished rats after LT. Similarly, after allograft LT in BN rats, Xie et al. [89] found that the numbers of *Lactobacillus* and *Bifidobacterium* in the probiotic group were significantly greater than the antibiotic and allograft groups. Liver injury was significantly reduced in the probiotic group compared with the allograft group. Moreover, the study revealed that probiotics mediated their beneficial effects through an increase of Treg cells and TGF-β and reduction of CD4/CD8 in rats with AR after LT [89].

Probiotic administration is also useful in ameliorating gut microbial dysbiosis after SBT. Compared with non-treated hosts, small bowel histological injuries were significantly ameliorated and BT was reduced in rats with 6 days of probiotics treatment after SBT [90]. Similarly, in mice with an allogeneic stem cell transplantation, modifying the intestinal microbiota using the probiotic microorganism *Lactobacillus rhamnosus* resulted in a reduced translocation of enteric bacteria to the mesenteric lymph nodes, reduced acute GVHD and improved survival [91].

## Conclusions and perspectives

With the improvement of transplantation techniques and postoperative recovery treatments, allogeneic transplantation has become an effective therapy for a variety of end-stage diseases. However, various postoperative complications, such as infection, rejection and GVHD, still restrict the use of allogeneic transplantation. In recent years, because of the progress in microbial detection technologies, many animal and human studies have shown that populations and diversity of gut microbiota are altered after allogeneic transplantation. When postoperative complications occur, gut microbiota populations and diversity are in a more significant dysbiosis. Furthermore, distinct gut microbial profiles could be potential diagnostic biomarkers of complications after transplantation [52, 61] and even predict a patient’s history before transplantation [64]. However, this needs to be confirmed in more studies. Whether microbial changes cause or follow complications after transplantation is still unclear.

In several studies, intervention methods, such as antibiotics, probiotics and prebiotics, could effectively regulate gut microbiota and reduce the incidence of complications after transplantation. Thus, gut microbiota has the potential to be a novel therapeutic target to restrict, improve and even reverse complications after allogeneic transplantation. However, more studies revealing the definite mechanism of these results are needed. The role of intestinal decontamination in allogeneic transplantation is still controversial. For example, several studies showed that selective perioperative intestinal decontamination did not reduce infectious complications after LT [83–85]. However, in HSCT, complete intestinal decontamination could significantly reduce the occurrence of postoperative GVHD [86].

Recently, the study of gut microbiota after allogeneic transplantation has significantly progressed. However, there are still many limitations that need to be resolved. Thus far, most studies evaluating gut microbial characteristics after allogeneic transplantation rely on rectal sampling only. However, the diversity and population of microbiota along the gastrointestinal tract are significantly different. Gu et al. [92] found higher phylogenetic diversity in gastric, duodenal, large intestinal and fecal samples than jejunum and ileum samples. Moreover, a greater proportion of anaerobes, such as Bacteroidaceae, Prevotellaceae, Rikenellaceae, Lachnospiraceae, and Ruminococcaceae, were found in the large intestine and feces. However, a larger proportion of Lactobacillaceae were found in the stomach and small intestine. Inferring the status of the whole gut microbiota only by rectal samples may be a challenge. Therefore, the gut microbial profiles along the intestine should be studied in the future. Many studies have been performed on rats. However, there are major differences in the gross anatomy, physiology and food processed in gastrointestinal tract between a mouse and human. Consequently, a host’s gut microbiota may be highly divergent in species, as well. Overall, two major phyla, Bacteroidetes and Firmicutes, account for the dominant gut microbiota in humans and mice [93]. However, in genera taxonomic classifications, 85 % of bacterial genera found in mouse gut microbiota was not present in humans [94]. Thus, to obtain more credible and relevant results, more human studies are needed.

Using metagenomics to investigate fecal samples from 124 European individuals, the MetaHIT consortium found more than one thousand microbial species in the human gut, and over 99 % of them were bacteria [1]. A core healthy human gut microbiome also has been explored and postulated to consist of three enterotypes, typified by the relative dominance of particular groups of organisms: *Prevotella*, *Ruminococcus* and *Bacteroides* spp. [95]. On average, individual microbiota could have long-term stability [96]. However, patients’ gut microbiota pre- and post-surgery [97] or drug consumption [98, 99] may be different. Gut microbiota can also be impacted by many other factors, including host genes [100], immune system [46], geography [101], age [102], weight [103], lifestyle [104], season [105] and diet [106]. For example, significant inter- and intra-individual variations in seasonal stabilities of the human gut microbiota were found [107]. In addition, due to the difference in long-term dietary habits, the human gut microbiome abundance and proportions varied between United States individuals [108]. David et al. [109] also found that short-term consumption of diets composed entirely of animal or plant products altered gut microbial community structure. Most transplant patients are put on special diet during hospitalization, and gut microbiota can be impacted by diet alone. Antibiotics are usually used after allogeneic transplantation, which also can lead to the alteration of gut microbiota [110]. In future studies, these factors should be taken into account.

Current studies showing a relationship between allogeneic transplantation and gut microbiota were mainly concentrated in liver transplantation, small bowel transplantation, kidney transplantation and hematopoietic stem cell transplantation. In addition, the role of a host’s immune system in the correlation of gut microbial dysbiosis and complications is rarely studied and is mostly limited to HSCT [111, 112]. Finding the definite microbiota effect on local and distal immune system that may lead to complications post-transplantation is important. Moreover, molecular pathways by which microbial signals can lead to complications after transplantation should be evaluated in future studies. Modulating infection and alloimmune responses after transplantation may become possible by identifying therapeutic targets. For example, in preclinical and clinical trials, agents that block TLRs are being tested to reduce pathology in septic or autoimmune patients [30]. These should be in-depth studies and extend to other types of transplantation.

## Abbreviations

LT: liver transplantation
OLT: orthotopic liver transplantation
SBT: small bowel transplantation
KT: kidney transplantation
HTCT: hematopoietic stem cell transplantation
IGVHD: intestinal graft versus host disease
iTregs: induced regulatory t cells
PCR-DGGE: polymerase chain reaction-denaturing gradient gel electrophoresis
NGS: next-generation sequencing
IFN: interferon
IL: interleukin
MHC: major histocompatibility complex
APCs: antigen-presenting cells
TNF: tumor necrosis factor
IECs: intestinal epithelial cells
IELs: intraepithelial lymphocytes
ILFs: isolated lymphoid follicles
PPs: Peyer’s patches
MLNs: mesenteric lymph nodes
IgA: immunoglobulin A
NKTs: natural killer T cells
TGF-β: transforming growth factor-beta
SFB: segmented filamentous bacteria
TCRs: T cell receptors
FoxP3: forkhead box protein 3
LPS: lipopolysaccharides
BT: bacterial translocation
AR: acute rejection
CR: chronic rejection
BMT: bone marrow transplantation
SDD: selective digestive decontamination
TGID: total gastro-intestinal decontamination
